# The Functional Implications of Endothelial Gap Junctions and Cellular Mechanics in Vascular Angiogenesis

**DOI:** 10.3390/cancers11020237

**Published:** 2019-02-18

**Authors:** Takayuki Okamoto, Haruki Usuda, Tetsuya Tanaka, Koichiro Wada, Motomu Shimaoka

**Affiliations:** 1 Department of Pharmacology, Faculty of Medicine, Shimane University, 89-1 Enya-cho, Izumo-city, Shimane 693-8501, Japan; h-usuda@med.shimane-u.ac.jp (H.U.); tetsu@med.shimane-u.ac.jp (T.T.); koiwada@med.shimane-u.ac.jp (K.W.); 2 Department of Molecular Pathobiology and Cell Adhesion Biology, Mie University Graduate School of Medicine, 2-174 Edobashi, Tsu-city, Mie 514-8507, Japan; shimaoka@doc.medic.mie-u.ac.jp

**Keywords:** gap junction, connexin, angiogenesis, cell mechanics, cell migration, cellular stiffness

## Abstract

Angiogenesis—the sprouting and growth of new blood vessels from the existing vasculature—is an important contributor to tumor development, since it facilitates the supply of oxygen and nutrients to cancer cells. Endothelial cells are critically affected during the angiogenic process as their proliferation, motility, and morphology are modulated by pro-angiogenic and environmental factors associated with tumor tissues and cancer cells. Recent in vivo and in vitro studies have revealed that the gap junctions of endothelial cells also participate in the promotion of angiogenesis. Pro-angiogenic factors modulate gap junction function and connexin expression in endothelial cells, whereas endothelial connexins are involved in angiogenic tube formation and in the cell migration of endothelial cells. Several mechanisms, including gap junction function-dependent or -independent pathways, have been proposed. In particular, connexins might have the potential to regulate cell mechanics such as cell morphology, cell migration, and cellular stiffness that are dynamically changed during the angiogenic processes. Here, we review the implication for endothelial gap junctions and cellular mechanics in vascular angiogenesis.

## 1. Introduction

Vasculogenesis leads to the creation of the heart and the first primitive vascular plexus inside the embryo and its surrounding membranes, as can be observed in yolk sac circulation. In contrast, angiogenesis is the physiological process of neovascular formation from pre-existing blood vessels during adult tissue homeostasis and tumorigenesis [1], and is responsible for the remodeling and expansion of the vascular network. Angiogenesis is a multi-step process that includes degradation of the basement membrane, vascular destabilization, angiogenic sprouting, endothelial tip cell migration, endothelial stalk cell proliferation, lumen formation by endothelial cells, and vascular stabilization [2]. Vascular endothelial cells covering the lumen of the blood vessel play a leading role in angiogenesis. In response to these pro-angiogenic and environmental factors, endothelial cells initiate angiogenic processes [3,4], which can be categorized as vascular sprouting [5], cell proliferation [6], cell migration [7], tube formation [8], and vascular stabilization [9]. Notably, during these angiogenic processes, endothelial cells dynamically alter cell mechanics, and physiological components determined by cytoskeletal rearrangement [10], focal adhesion formation [11], and contractile force [12], have also been observed. 

The vascular network, which supplies oxygen and nutrition, is necessary for tumor growth and cancer cell proliferation. Anti-tumor angiogenic therapy has shown some promise in the treatment of several cancers [13]. Although current anti-angiogenic therapies reduce the density of tumor blood vessels and tumor size, they might create a hypoxic and acidic tumor microenvironment in tumor tissues, which could induce cancer cells to become more aggressive and metastatic [14,15]. Moreover, it has been reported that tumor vascular normalization facilitates drug and immune cell delivery, resulting in decreased tumor size and reduced metastasis [16]. New anti-cancer therapies that target tumor angiogenesis focus on controlling aberrant angiogenesis and vascular normalization [17,18]. A better understanding of angiogenesis is needed to achieve high efficacy in anti-cancer therapies. 

Gap junctions consist of connexin (Cx) family protein, which has four transmembrane domains and two extracellular loop domains [19,20]. The amino and carboxyl terminal of Cx protein are located on the cytoplasmic side of the membrane. The C-terminal domains are highly variable among the family members and have several phosphorylation sites that transmit signals in order to control the opening and closing of channels [21,22]. The hexameric Cx forms a hemichannel (connexon) that docks to another connexon on the adjacent cell via extracellular domains resulting in the formation of gap junction channel [19,20]. Gap junction channels directly connect each cytoplasm of adjacent cells, and in this manner contribute to both electrical and biochemical coupling. Electrical coupling plays a role in the generation of highly synchronized electrical activity [23]. In contrast, biochemical coupling allows for the intercellular movement of small molecules and metabolites [24]. Thus, gap junction intercellular communication is essential for the transfer and synchronization of the intracellular environment between adjacent cells. It is though that the gap junction-mediated transfer and synchronization of intracellular mediators such as ions, amino acids, small metabolites, and secondary messengers are essential in orchestrating multicellular responses [24]. In addition, the C-terminal domain of Cx protein interacts with several intracellular proteins such as signaling molecules [25], cytoskeletal proteins [26], and cell junctional proteins [27], indicating the possibility that gap junctions and Cx proteins mediate the regulation of cell mechanics and mechanotransduction. 

Post-translational modification of Cxs carboxyl terminal residues play an important role in the regulation of the Cx protein life cycle to include oligomerization, trafficking, gap junction formation, gating function, and internalization [28,29,30]. Cx43 is phosphorylated soon after synthesis and phosphorylation changes as it traffics to the plasma membrane, ultimately forming gap junction structures [28,31]. Cx43-serin364 (S364) phosphorylation and Cx43-S325/S328/S330 phosphorylation increase gap junction assembly and stability, resulting in the enhancement of gap junction intercellular communication [32,33]. Cx43-S368 phosphorylation by protein kinase C decreases gap junction communication in vascular wall cells [34,35]. Furthermore, Cx43-S255/S279/S282 phosphorylation by mitogen-activated protein kinase also reduces gap junction communication by inhibiting the channels from opening [36,37]. In addition to phosphorylation, other modifications to Cxs, such as nitrosylation and ubiquitination, have been reported [29,38]. The ubiquitin proteasome system might contribute to the internalization of gap junctions and degradation of Cxs [29]. It has been reported that Cx43 is probably polyubiqutinated and that the proteasomal inhibitor induces increased and prolonged Cx43 expression [39]. Inhibition of Cx43 and ubiquitin proteasome-related proteins alters the Cx43 and ZO-1 interaction, leading to an increase in gap junctions at membranes [40]. These studies thereby suggest that the life cycle of gap junctions and Cx hemichannels is tightly regulated by a balance of post-translational modifications to Cx protein.

Endothelial cells play a critical role in regulating vascular inflammation [41], blood coagulation [41,42], leukocyte adhesion and extravasation [43], and angiogenesis [44]. Thus, endothelial cell dysfunction is a conceivable cause of the onset of cardiovascular diseases [42]. Endothelial cells predominantly express three Cxs: Cx37, Cx40, and Cx43 [45,46]. In addition, Cx32 and Cx45 are also detected in endothelial cells [47,48]. Interestingly, endothelial cells dynamically regulate gap junction function and Cx expression in response to pro-inflammatory stimuli [46,49,50]. Conversely, alteration of gap junction function and Cx expression in endothelial cells can impact multiple endothelial cell functions under physiological and pathological conditions [46,51,52]. More than a decade of research on gap junctions in endothelial cells and angiogenesis has produced evidence of the interplay between endothelial Cxs and angiogenesis. Recent studies have indicated that gap junctions and Cxs in endothelial cells contribute to several important steps in angiogenesis such as sprouting, migration, tube formation, and cellular stiffness, all of which are implicated in cellular mechanics. Here, we mainly focus on gap junctions and Cxs in endothelial cells and will discuss the implications of cellular mechanics for vascular angiogenesis.

## 2. Endothelial Cx Expression and Its Role in Vascular Diseases

Cx expression pattern in endothelial cells is dependent upon vessel type, be it arteries, veins, or lymphatic vessels. Cx37 and Cx40 are co-expressed in the arterial endothelial cells of healthy vessels [53,54], whereas Cx43 has been characteristically observed in endothelial cells of the microvasculature and at branch points of arteries subject to turbulent blood flow [54,55]. Cx32, Cx37 and Cx40 are abundantly presented in venous endothelial cells [47,56,57]. Cx43 is mainly located in the medial layer of arteries and is detected in rat and human veins at low levels [56,57]. In vitro studies have demonstrated Cx32, Cx37, Cx40, and Cx43 expression in both cultured human vein and artery endothelial cells [58,59,60]. It has been known that alterations to Cx expression and gap junction function in endothelial cells upon pro-inflammatory stimuli is closely correlated with endothelial cell activation. Indeed, pro-inflammatory tumor necrosis factor-α reduces gap junction function in endothelial cells at an early phase (4 h) and then decreases the expression of Cx32, Cx37 and Cx40, but not Cx43 during the late phase (24 h) [50,61]. Lipopolysaccharide, an important activator of inflammation in endothelial cells via toll-like receptor 4, also induces serine-dephosphorylation of Cx40 [62] and reduces gap junction function between microvascular endothelial cells [62]. In addition, lipopolysaccharide diminishes gap junction intercellular communication between microvascular endothelial cells through tyrosine phosphorylation of Cx43 [63]. Pro-coagulant factor thrombin, which is a major trigger of thrombus formation and increased vascular endothelial permeabilization, induces rapid and acute internalization of Cx43-mediated gap junctions in primary pulmonary artery endothelial cells [64]. However, opposite effect, whereby thrombin induces Cx43 expression and gap junction function associated with disruption of the endothelial barrier has also been reported [65]. In this way, although different phenotypes have been observed, these results demonstrate the dynamic regulation of gap junction function and Cx expression in endothelial cells upon pro-inflammatory stimuli at both the post-translational modification and transcriptional level.

Several studies have shown that aberrant gap junction function and Cx expression in endothelial cells contributes to the promotion of endothelial dysfunction and vascular inflammatory diseases such as atherosclerosis. For example, Cx37 and Cx40 are found at lower levels during the early stage of atherosclerosis [46], while deletion of Cx40 from endothelial cells in mice, as well as the dysfunction of Cx37, can promote the development of atherosclerosis by enhancing both monocyte adhesion and transmigration [51,66]. Moreover, Cx37-deficient mice enhance the expression of a number of pro-inflammatory genes involved in advanced atherosclerosis [67]. Cx43 is increased in the early stage of atherosclerosis [46], whereas reduced expression of Cx43 by smooth muscle cells inhibits the formation of atherosclerotic lesions [68]. Furthermore, endothelium-specific deletion of Cx43 modulates renin secretion, thereby inducing hypertension [69]. A Cx43 mutation in patients with cardiac infarction has been identified as a risk factor [70]. We have previously shown not only that reduced Cx32 expression in human umbilical vein endothelial cells (HUVECs) facilitates pro-inflammatory cytokines expression upon inflammation [61], but also that Cx32-deficient mice experience enhanced activation of vascular inflammation and blood coagulation in a model of sepsis [61,71]. It has been reported that ageing-related downregulation of Cx43 in atrial tissues in old guinea pigs facilitates the development of atrial fibrillation [72]. Taken together, these studies suggest that abnormalities in gap junction function and Cx expression may act as a trigger for various endothelial dysfunctions, leading to the development of atherosclerosis and vascular inflammatory diseases [49]. 

## 3. Alterations of Gap Junction Function and Cx Expression in Endothelial Cells in Response to Pro-Angiogenic Stimuli

**Figure 1:**
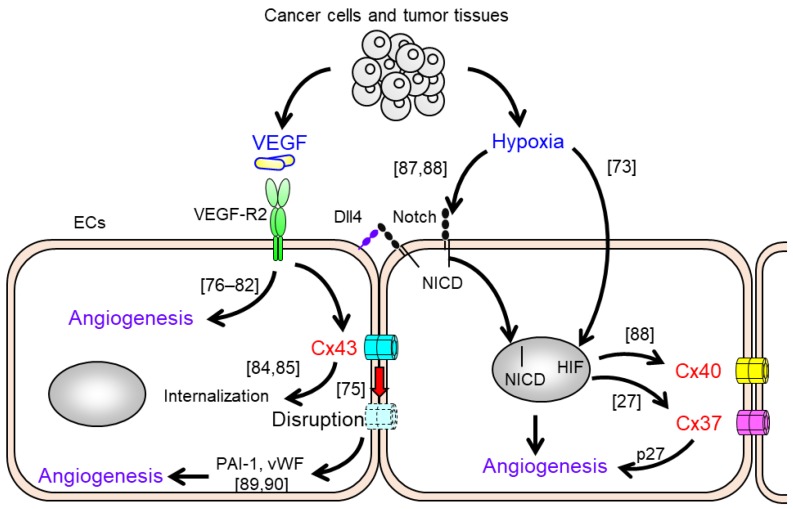
Alteration of gap junction function and Cx expression in endothelial cells (ECs) in response to pro-angiogenic stimuli. Vascular endothelial growth factor (VEGF), secreted by cancer cells, is an essential initiator of angiogenesis [82]. Endothelial cells induce internalization and disruption of gap junctions (GJs) formed by Cx43 under VEGF-VEGF-R2 signaling [75,84,85]. The impairment of Cx43 increases proangiogenic plasminogen activator inhibitor-1 (PAI-1) [89] and von Willebrand factor (VWF) [90]. Hypoxic conditions in tumor tissue activate Notch and hypoxia-inducible factors (HIFs) in endothelial cells [73,87,88]. Notch signaling including the nuclear translocation of the notch protein intracellular domain (NICD) induces endothelial cell function and cell mechanics involved in angiogenesis. HIF pathways play an important role in the induction of angiogenic-related genes expression in endothelial cells [73]. Both signaling pathways result in angiogenesis becoming associated with upregulation of Cx37 and Cx40 in endothelial cells [27,88].

Pro-angiogenic factors that are released from tumor tissues and cancer cells [73,74], are also been thought to modulate the gap junction function and Cx expression of endothelial cells [75] (Figure 1). Vascular endothelial growth factor (VEGF), which plays a central role in both vasculogenesis and angiogenesis [76], has implicated in diverse physiologic processes including tumor angiogenesis [77,78], diabetic retinopathy [79], wound healing [80], and tissue repair following ischemic injury [81]. VEGF-induced VEGF-receptor 2 (VEGF-R2) activation of endothelial cells in existing vasculature is primarily an initial step of angiogenesis, which then leads to sprouting, cell proliferation, and cell migration of endothelial cell [82]. In in vitro model experiments, VEGF-induced c-Src tyrosine kinase and mitogen-activated protein kinase activation results in the rapid disruption of gap junction function in endothelial cells [75], and increases the paracellular endothelial permeability associated with reduction in cell-cell junctions [83]. Furthermore, it has been reported that the VEGF-induced disruption of gap junction function correlates with the rapid internalization of Cx43 and Cx43 tyrosine phosphorylation in rat coronary capillary endothelium [84,85]. Therefore, pro-angiogenic VEGF stimulation negatively modulates gap junction function and Cx expression in endothelial cells as a consequence of angiogenesis-related signaling.

In addition to VEGF, basic fibroblast growth factor (bFGF) and hypoxia are well known as a pro-angiogenic factor and a conducive environment. It has been reported that microvascular endothelial cells facilitate gap junction function and Cx43 expression in response to bFGF stimulation [86]. Stimulation with bFGF not only increases Cx43 mRNA expression but also facilitates Cx43 localization at the cell-cell interface [86]. The hypoxia conditions observed in tumor tissue activate hypoxia-inducible factor (HIF) pathways and induces the expression of a number of pro-angiogenic genes in cancer cells [73]. In the case of endothelial cells, hypoxia upregulates Notch ligand Dll4 expression and promotes activation of Notch signaling, which is an essential pathway for vascular development and stabilization [87,88]. The upregulation of Cx40 expression has been reported under hypoxia-mediated Notch signaling in endothelial cells [88]. One recent study has shown that a Notch-Cx37-p27 axis promotes endothelial cell cycle arrest, leading to vascular regeneration under shear stress [27]. These results suggest that endothelial Cx and Notch signaling might coordinate the appropriate endothelial cell proliferation and angiogenesis.

Endothelial gap junction function and Cx expression are assuredly regulated by pro-angiogenic and environmental factors. It has also been reported that Cx expression and gap junction function in tumor cells [91], myocadiac cells [92], and mesenchymal stem cells [93] are tightly linked to VEGF expression in these cells. For example, Cx43 knock-down in tumor cell lines increased VEGF expression and enhanced the proliferation of endothelial cells [91]. Recent studies have shown Cx43 to be present in exosomes [94], which are extracellular small vesicles that carry various bioactive molecules, such as enzymes, metabolites, eicosanoids, and small RNAs. Shimaoka and colleagues have summarized the biological role of Cxs in exosomes [95]. It is interesting to speculate that the anti-angiogenic microRNAs in exosomes, which are instantaneously delivered via Cx channels, might inhibit the promotion of angiogenesis by cancer cells [96]. Thus, in order to understand the role of gap junctions and Cxs in angiogenesis, it is first necessary to elucidate the basic biology of gap junction and Cx in these types of cells at the interplay of angiogenesis and tumor development.

## 4. The Impact of Endothelial Cxs on Vascular Endothelial Angiogenesis

Several groups have investigated the impact of Cxs on the development of the cardiovascular system, which is closely related to angiogenesis. Mutations in the gene for Cx43 (GJA1) were found to cause a hypoplastic left-heart syndrome [97]. Cx43-deficient mice, which die at birth from congenital heart malformations, have shown a reduction in the distal branching complexity and length of their coronary arteries [98]. In Cx40-deficient mice, cardiac malformations have also been observed [99]. Additionally, both endothelial Cx40- and Cx37-knockout mice develop severe abnormalities of the vascular function and structure [100]. Recently, loss of endothelial Cx40 was found to lead to a reduction in vascular growth and capillary density in the neovascularization of the mouse neonatal retina [101]. We have also demonstrated that aortic vascular tissue from Cx32-deficient mice exhibits suppressed vascular sprouting of endothelial cells [59]. Cx37 knock-out mice enhance vasculogenesis and remodeling allowing improvement from an ischemic hindlimb injury [102]. These studies indicate the contribution of endothelial Cxs to angiogenesis under physiological or pathological conditions.

Some reports have shown the relevance of endothelial Cxs expression and vascular angiogenic potential in endothelial cells using in vitro angiogenesis assay. Knockdown of Cx43 using specific siRNAs reduces tube formation and cell proliferation of human aortic endothelial cells [103]. The downregulation of Cx43 increases angiogenesis-related factors [103], such as plasminogen activator inhibitor-1 [89] and von Willebrand factor [90]. In addition, alterations in endothelial Cx43 expression in mice inversely correlate with VE-cadherin expression and microvessel permeability [104], which is induced by acute inflammation and pathologies associated with angiogenesis. These include tumors, wounds, and chronic inflammatory diseases. Such studies suggest that endothelial Cxs might directly and/or indirectly contribute to angiogenesis through the modification of endothelial cell function. Knockdown of Cx37, Cx40, or Cx43 using siRNAs has been shown to suppress endothelial angiogenesis including the branching of HUVECs, elongation of cell length, and tube formation by an in vitro Matrigel assay [105]. In gain-of-function experiments utilizing stable Cx-transfectants, we have demonstrated that increased expression of Cx32 markedly enhances tube length and the number of branching EA. hy926 cells [106], which are an endothelial cell line derived by fusing HUVECs with a human lung carcinoma A549 cell during Matrigel tube formation [59]. On the other hand, Cx37- or Cx43-transfected EA. hy926 cells impair tube length and the number of branching [59].

While these studies have provided extensive evidence that endothelial Cx expression modulates angiogenesis, the specific impact of each Cx on angiogenesis remains unclear. Notably it is believed that any endothelial Cx expression may modify the expression of other Cxs [59,105,107]. Indeed, Cx43 siRNA induces increased expression of both Cx37 and Cx40 in aortic endothelial cells. In HUVECs, Cx43 siRNA does not alter the expression of other Cxs, whereas Cx40 siRNA and Cx37 siRNA reduce Cx43 and Cx40 expression, respectively [105]. In addition, Cx32-transfected EA. hy926 cells reduce Cx43 expression and have exhibited highly angiogenic potential, such as in tube formation and branching [59]. Although gain-of function and loss-of function assays have yet to be experimentally tested, there is evidence that alterations in Cx expression patterns and their relevant network of Cx expression may elicit different endothelial cell phenotypes during angiogenic processes. Several mechanisms by which specific Cxs regulate inflammation, coagulation, cell migration, and proliferation, have been proposed. Thus, the possibility has been suggested that novel predominant Cxs resulting from target Cx manipulation might influence angiogenesis on behalf of these manipulated Cxs. This interrelated Cx regulatory network has made it difficult to understand the specific role of each endothelial Cx in angiogenesis.

## 5. Endothelial Cx-Dependent Regulation of Cell Migration in Angiogenesis

**Figure 2:**
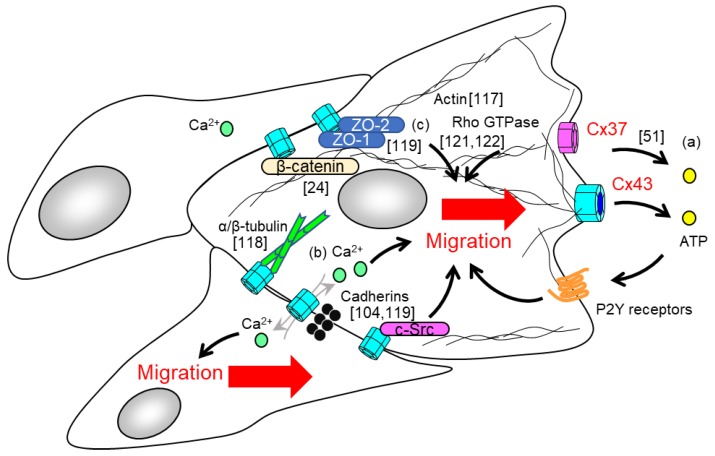
Endothelial Cx-dependent regulation of cell migration in angiogenesis. Gap junctions and Cxs regulate cell migration via channel function dependent and independent pathways. (**a**) Extracellular ATP released by Cx-hemichannels activates P2Y receptors, which trigger cell migration [48]. (**b**) Gap junction-mediated propagation of calcium waves is required for collective cell migration. (**c**) The interaction of Cx and gap junction with cytoskeletal proteins or intracellular proteins orchestrates cytoskeletal rearrangement and cell migration [24,116].

Endothelial cells dynamically alter cell mechanics such as cell morphology, cell proliferation, and cell migration during angiogenesis [108,109]. Endothelial cell activation by pro-angiogenic factors allows tip cells to extend filamentous actin (F-actin)-rich filopodial protrusions that migrate toward the required site [2,3,110]. Tip cells are the leading cells of the sprouts and guide the ensuing stalk cells, which proliferate in order to elongate the sprout [4]. Fine tuning of migrating tip cells and proliferating stalk cells is crucial for angiogenesis [4]. Notably, endothelial Cxs have been increasingly implicated in the control of endothelial cell migration. We have shown impaired cell migration of endothelial cells both in an in vitro wound healing assay (via blocking Cx32 in endothelial cells) and in an in vivo Matrigel plaque implant assay in Cx32-deficient mice [59]. Other groups have reported that gap junction intercellular communication and Cx43 expression are increased in the region of cell migration and at the wound edge by using a wounded monolayer repair assay [111]. In a wound assay, Cx43 expression in immortalized endothelial cells was positively associated with cell migration and wound closure [112]. Cx43-specific siRNA markedly suppresses cell migration of endothelial progenitor cells, as demonstrated by a Transwell chamber migration assay, which allowed cells to migrate through the filter membrane upon being stimulated with pro-angiogenic factors [113]. Although endothelial Cx-dependent regulation of cell migration has been experimentally tested, it remains to be seen whether this mechanism functions in the same manner under physiological condition. In addition to endothelial cells, several types of cells such as leukocytes, epithelial cells, and tumor cells also regulate their migration via gap junction channel dependent and independent function (reviewed by Matsuuchi [114] and Kameritsch [115]). Both gap junction mediated cell-cell interactions and hemichannel functions are involved in the regulation of cell migration in a number of cell types (Figure 2). These studies proposed the concept of gap junction- and Cx-mediated cell migration and provided an overall better understanding of endothelial cell migration [114,115].

The intracellular domain of Cx protein interacts with other proteins to aid the structural stability of cell-cell junctions sustained by cytoskeletal scaffolds [24]. Due to the ubiquitous distribution of Cx43, many studies have focused on Cx43 and its interacting proteins [116]. The carboxyl tail of Cx43 does indeed interact with several cytoskeletal proteins such as F-actin [117], α-/β-tubulins [118], cadherins [119], and cortactin [120]. For example, the membrane expression of N-cadherin or of ZO-1 is dominantly localized in the existing site of the Cx43 protein [119]. The interaction of Cx43 with the cadherin family may be important not only for the mechanics of cell migration, but also for the generation of intracellular signaling. Interaction of Cx43 and cadherins coordinates activation of Rho GTPases, which promote cell motility and invasion [121,122]. Moreover, Rac1 in migrating cell is dominantly found in actin-rich structures, which in conjunction with E-cadherin, is believed responsible for generating the traction forces of germ cells in vivo [123]. As the intracellular carboxyl tail of Cx43 has a number of interaction partners, the transfection of a mutant Cx43 lacking the intracellular carboxyl tail impairs cell migration [124]. Cx43 deficiency causes impaired polarization due to the non-directional alignment of the microtubule organizing center. This results not only in a loss of directionality of cell migration, but also in impaired development of coronary arteries, as can be observed in Cx43 deficient mice. An epicardial cell that expresses the Cx43 mutant, but which lacks a tubulin-binding site in the carboxyl tail, exhibits a phenotypic pattern similar to a cell lacking Cx43 [124]. These suggest that the interaction between Cx43 and cytoskeletal protein may coordinate cellular mechanics and behavior.

## 6. Potential Role of Endothelial Cellular Stiffness in Cell Migration

The interaction between Cx and cytoskeletal proteins contributes to the regulation of cellular stiffness which is defined as the physical property of a cell to resist deformation in the response to any applied force. A contraction force generated by the actomyosin cytoskeleton and F-actin has been inseparably linked to the regulation of cellular stiffness [125,126]. Activation of the Rho-actomyosin signaling pathway enhances the formation of actin bundles, stress fibers, and tensile actomyosin structures [127], all of which correlate with cellular stiffness [128,129]. Thus, interplay between endothelial Cxs and the Rho family has been implicated in the regulation of cellular stiffness. We have found that proinflammatory stimulation increased the endothelial cellular stiffness associated with impaired gap junction function, cytoskeletal remodeling, and focal adhesion formation [130]. Moreover, blockade of gap junctions induces the cellular stiffening associated with focal adhesion formation and cytoskeletal rearrangement and prolongs tumor necrosis factor-α-induced endothelial cellular stiffening [130]. This study has provided the first evidence that endothelial gap junction contributes to the regulation of endothelial cellular stiffness via interaction with cytoskeletal rearrangements.

It is thought that endothelial cellular stiffness may be a determinant factor of leukocyte adhesion to endothelium [131]. In general, leukocytes sense the stiffness of the extracellular substrate via integrin-ligand interactions and adhere more strongly to stiff substrates [132]. Endothelial cells materially work as a substrate during leukocyte adhesion and migration. Leukocyte integrins assume both selective and cohesive adhesion via binding to distinct endothelial adhesion receptors such as the intercellular adhesion molecule 1 (ICAM-1) [125]. More recently, van Buul and colleagues have demonstrated that endothelial stiffening helps stabilize ICAM-1 adhesome in order to promote leukocyte spreading [133]. Integrins increase the binding avidity to ligands, which correlates with endothelial cellular stiffness. In addition, the integrin-focal adhesion complex generates contractile forces in cells and transduces these forces into a mechanosignaling complex [131,134]. These suggested the possible mechanism which regulates leukocyte adhesion and activation via physical endothelial cellular stiffness [135].

**Figure 3:**
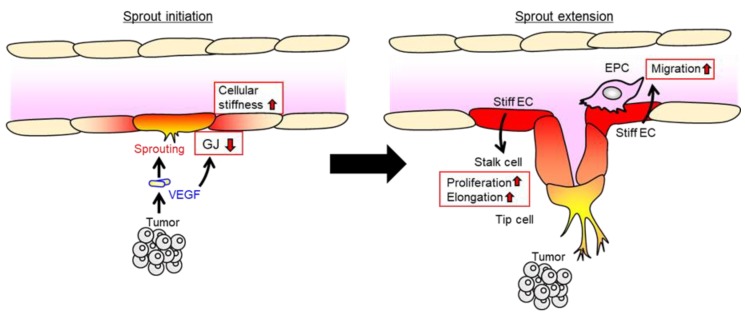
Potential role of endothelial cellular stiffness in cell migration. VEGF induces gap junction (GJ) reduction and the sprouting of endothelial cells may result in the stiffening of endothelial cells (ECs) during the sprout initiation phase. Stiff endothelial cells can work as a substrate for attached surrounding cells, whereas, provide a favorable environment for the recruitment of endothelial progenitor cells (EPCs), and then also support adjacent stalk cell proliferation and elongation.

In addition to leukocytes, endothelial cells themselves have been shown to modulate their own migration, proliferation, and morphological changes in response to extracellular substrate stiffness [136,137]. In the case of lymphatic vessel formation, a soft extracellular matrix might control lymphangiogenesis through the induction of GATA2 expression and its related genes, which are involved in cell migration and lymphangiogenesis, including VEGF-R3 and Cx37 [138]. Thus, it is possible that stiffening endothelial cells in the existing vasculature are favorable to the recruitment of pro-angiogenic tip cells and stalk cells at the sprouting spots (Figure 3). Of note, VEGF-induced cytoskeletal rearrangement and impaired gap junction function might be supposed to increase endothelial cell stiffness. Stiff endothelial cells may recruit endothelial progenitor cells and support the cell proliferation and elongation of stalk cells. Taken together, a series of studies makes the case that gap junction-mediated endothelial cell stiffening may facilitate the angiogenic process of endothelial cell recruitment by activating a mechanosensing and transduction pathway.

## 7. Conclusions and Future Perspectives

We are beginning to understand that gap junction and Cx in endothelial cells might serve as a connection center between biological function and cell mechanics in the context of angiogenesis. In this review, we provided an overview of the endothelial gap junction function and Cxs expression found under pro-angiogenic conditions and the functional role of endothelial gap junctions and Cxs in cell mechanics during the angiogenic process. Although several studies have demonstrated gap junction-/Cx-dependent regulation of angiogenesis, these mechanisms are still speculative and controversial. Notably, analysis of alterations to gap junction functionality and Cx expression patterns in pro-angiogenic stimuli in neovasculature are needed in order to properly characterize these de novo blood vessels involved in tumor angiogenesis. The involvement of gap junction and Cx in the regulation of cellular stiffness and cellular mechanics via their interaction with intracellular molecules has been shown; however, the dual roles of Cx as a mechanosensor and mechonotransducer remain unclear. Cellular stiffness- and mechanics-based mechanisms hold promise of helping us better understand the physiological and pathological components of angiogenesis and endothelial cell functionality. Additionally, gap junction- and Cx-mediated cell-cell interactions in a number of other cell types such as vascular smooth muscle cells, pericytes, fibroblasts, macrophages, and tumor cells also contribute to tumor angiogenesis through the expression of pro-angiogenic factors. Thus, further studies on the basic biology of gap junctions and Cxs in these type cells are needed for further elucidation, with a particular emphasis on the interplay of angiogenesis and tumor development. We speculate that gap junction and Cx targeting approaches may be relevant to the development of new treatments for cancer patients.
